# Dopamine Supersensitivity Psychosis Resolved With Electroconvulsive Therapy (ECT) in a Patient With Treatment-Resistant Schizophrenia: A Case Report

**DOI:** 10.7759/cureus.93361

**Published:** 2025-09-27

**Authors:** Sayui Takano, Tasuku Hashimoto, Ryunosuke Hayatsu, Nobuhisa Kanahara, Michiko Nakazato

**Affiliations:** 1 Department of Psychiatry, International University of Health and Welfare Narita Hospital, Narita, JPN; 2 Department of Psychiatry, Graduate School of Medicine, Chiba University, Chiba, JPN; 3 Division of Clinical Neuroscience, Chiba University Center for Forensic Mental Health, Chiba, JPN

**Keywords:** antipsychotic, dopamine d2 receptor, dopamine supersensitivity, schizophrenia, stimulation method

## Abstract

In individuals with schizophrenia, high-dose antipsychotic medication can induce an upregulation of the brain's dopamine D2 receptors (D2Rs), leading to dopamine supersensitivity (DS) or dopamine supersensitivity psychosis (DSP). For patients with established DSP, a reduction in the increased D2R density is sought, which in theory will restore the patients' therapeutic responsiveness to antipsychotics. We describe the case of a 50-year-old Japanese woman with schizophrenia who fulfilled the criteria for DSP and recovered from DSP after undergoing electroconvulsive therapy (ECT). Before the ECT, she had exhibited serious psychotic symptoms requiring admission and was treated with the chlorpromazine (CPZ) equivalent dose of 1,250 mg. After the ECT, she achieved a >2-year period of remission with a 35% reduction in the dose of the antipsychotic. Considering that ECT leads to D2R downregulation, the reduction in this patient's maintained antipsychotic dose may reflect the normalization of increased D2R density, resulting in the resolution of the DS state by ECT. This patient's case indicates that for individuals with schizophrenia and DPS, the application of ECT may effectively restore the therapeutic response to antipsychotics.

## Introduction

The age range that has shown the most frequent onset of schizophrenia is from later adolescence to early adulthood. Individuals with schizophrenia present a variety of psychiatric symptoms that may include positive symptoms (e.g., delusions and hallucinations), negative symptoms (e.g., affective flattening and avolition), and/or cognitive impairments. Unfortunately, the majority of individuals with schizophrenia experience a relapse of psychosis even if they have successfully achieved remission. The responsiveness of such relapse episodes to antipsychotic medication is greatly reduced compared to the responsiveness to first-episode psychosis (FEP), resulting in the need for a higher dose of antipsychotics (including polypharmacy) to control psychosis [1]. However, a long-term excessive blockage of the brain's dopamine D2 receptors (D2Rs) by a high-dose antipsychotic medication can increase the density of D2Rs (i.e., the blockage of up-regulated D2Rs), which may lead to the antipsychotic-induced dopamine supersensitivity (DS) state [2]. The DS phenomenon has traditionally been investigated based on receptor biochemistry (i.e., D2R up-regulation) or behavior (hyperlocomotion) in rodent models [3].

Dopamine supersensitivity psychosis (DSP) emerges in some patients with schizophrenia who reach the DS state. DSP is characterized by (i) rebound psychosis (i.e., relapse episodes immediately following the reduction, withdrawal, or switching of one or more ongoing antipsychotic agents), and/or (ii) acquired tolerance to the effects of antipsychotics (i.e., the antipsychotics no longer control the patient's psychotic symptoms, even at an increased dose). Neurological signs of the DS state include tardive dyskinesia and withdrawal dyskinesia [4,5]. Both the DS and DSP are related to treatment-resistant schizophrenia (TRS), which is generally defined as a failure to achieve a sufficient improvement of psychosis after the introduction of at least two different non-clozapine antipsychotics with sufficient doses and durations.

It has been estimated that DSP occurs in approximately 50%-70% of patients with TRS, despite the observation of their FEP's better response to the patients' initial medications [6,7], confirming that the development of DS or DSP is burdensome in patients with schizophrenia. Resolving the developed DS state and restoring patients' responsiveness to antipsychotic agents is thus an important issue in the management of patients with schizophrenia.

Electroconvulsive therapy (ECT) is indicated for a patient's severe psychomotor excitement or depressive state that is difficult to manage with pharmacotherapy. The mechanisms that underlie the effects of ECT are largely unknown, but several neurotransmitter systems are presumed to be involved. Experimental studies using rodent models and clinical investigations revealed that ECT has the potential to reduce the expression of D2Rs in the brain [8-10]. However, only a few investigations have examined the resolution of DSP by the application of ECT in clinical practice [11,12]. We present the case of a middle-aged woman diagnosed with schizophrenia who fulfilled criteria for both the DSP and TRS and recovered from DSP with the use of ECT, achieving a reduction of the necessary antipsychotic doses. We propose that ECT is a promising option for treating patients with both DSP and TRS, whose conditions are generally considered to be appropriate for treatment with clozapine.

## Case presentation

A 50-year-old Japanese woman had experienced auditory hallucinations and delusions of erotomania and persecution that had manifested as grossly strange and disorganized behavior since she was 28 years old. Her hallucinations included those indicated by her statements such as “My mother is not actually my mother,” “I am married to a celebrity. I always hear his voice cheering me,” and “I am pregnant with his child.” She had no reported family history of psychiatric diseases. She was first admitted to a hospital at 28 years old because her psychotic symptoms had acutely exacerbated. During her first hospitalization, she was treated with an antipsychotic medication (details unknown) and was discharged one month later with a remission of positive symptoms. She remained in remission with continuous hospital visits and antipsychotic medication for five years.

At 33 years of age, she suddenly discontinued the medication based on her own judgment. Within a month after discontinuing the medication, she experienced a relapse of severe psychotic symptoms and was secondarily admitted to another hospital; there, she was diagnosed with schizophrenia and began a regimen of 9 mg of haloperidol and 75 mg of levomepromazine, with a chlorpromazine (CPZ) equivalent dose (CP-eq.) at 525 mg. Her therapeutic response to this antipsychotic treatment was very good, and she remained in remission without positive symptoms for nine years of treatment while this regimen was continued.

At the age of 42 years, although the patient had continued to take her medications and reported no stressors, she relapsed and experienced positive symptoms with auditory hallucinations and persecutory delusions. Despite a change in antipsychotic medication to 3 mg risperidone, 2 mg brexpiprazole, and 85 mg levomepromazine (CP-eq. 785 mg), the symptoms did not improve. Moreover, negative symptoms including emotional and social withdrawal became apparent.

Five years later (age 47 years), the patient's psychotic symptoms (including delusions of pregnancy with a fictitious man, severe auditory hallucinations, and bizarre behaviors) were exacerbated. Although the long-acting injectable paliperidone palmitate (P-LAI) was introduced with increasing doses of the above-mentioned oral antipsychotics, the patient's symptoms did not improve (150 mg P-LAI, 2 mg brexpiprazole, and 275 mg levomepromazine (CP-eq. 1,475 mg)).

Two years later, the patient was admitted to a third hospital (age 49 years) because she repeatedly disappeared in order to meet with her fictitious husband. Although she underwent pharmacological treatments in various antipsychotic trials, her symptoms did not improve. The switch to aripiprazole worsened her psychosis, and thus, the drug regimen that had been administered earlier was resumed. Her therapeutic response to this regimen was very poor compared to that during her first psychotic episode. Consequently, her treatment was revised to high-dose antipsychotics: 150 mg P-LAI, 80 mg blonanserin patches (which is the maximum daily dose), and 50 mg levomepromazine (CP-eq. 1,250 mg).

**Figure 1:**
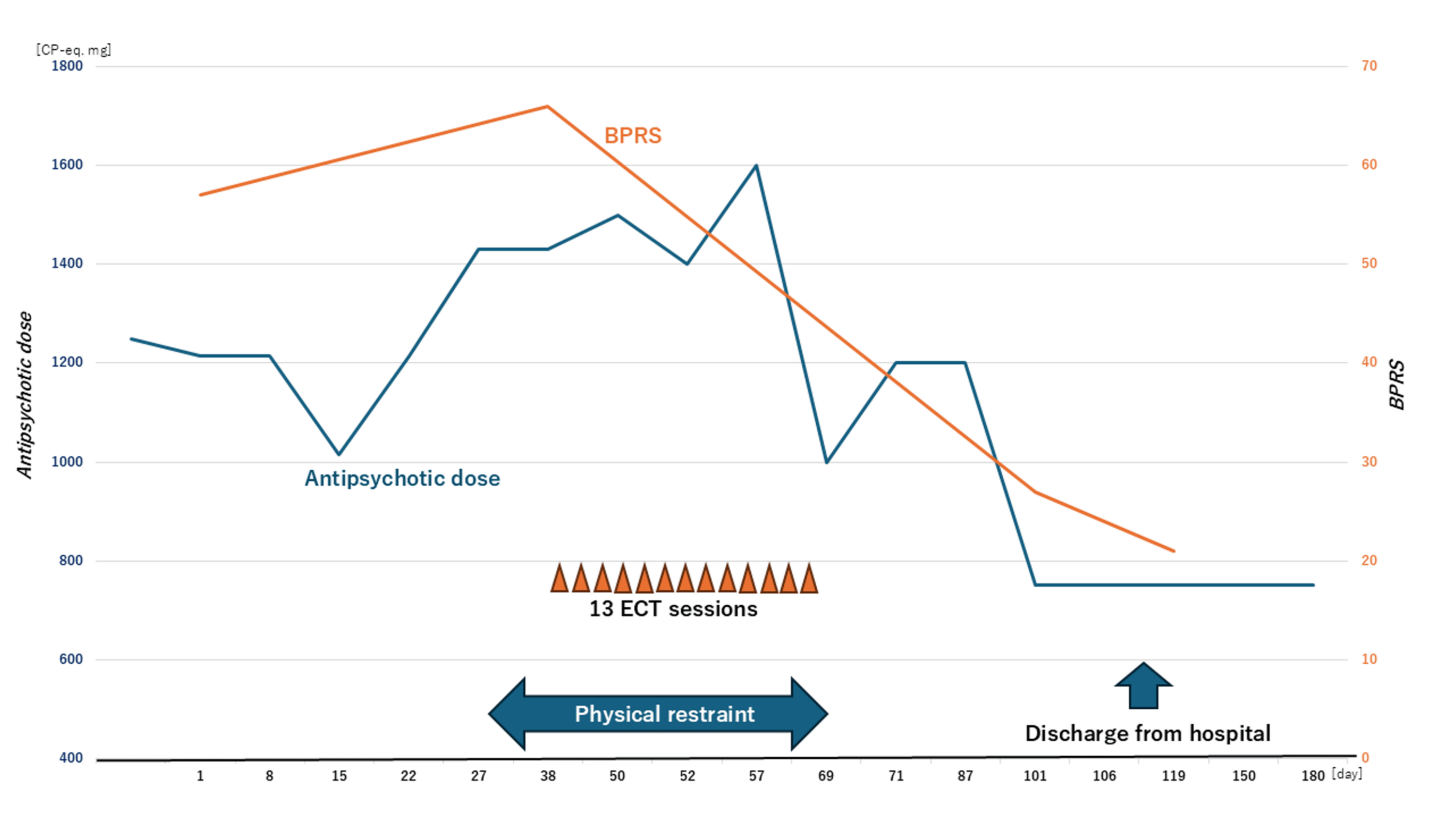
Changes over time in the patient’s chlorpromazine equivalent dose and her Brief Psychiatric Rating Scale (BPRS) scores after hospital admission. ECT: Electroconvulsive therapy

Approximately one year later (age 50 years), the patient was transferred to our hospital (her fourth admission) for intensive treatment, including ECT. Since she had exhibited serious psychotic symptoms including grossly disorganized and catatonic behaviors and severe auditory hallucinations (during which she was ‘ordered to face the wall’ during the examination) and refused to be hospitalized, she was involuntarily admitted to our hospital. As illustrated in Figure 1, on the day of admission her Brief Psychiatric Rating Scale (BPRS) score (18 items, with a 1- to 7-point scale for each item) was 57 points (positive symptoms, 18 points; negative symptoms, 16 points) as she was being treated with the above-mentioned regimen, i.e., 150 mg P-LAI, 80 mg blonanserin patches, and 50 mg levomepromazine (CP-eq. 1,250 mg).

After the patient's admission to our hospital, the levomepromazine was discontinued and the dosage of the blonanserin patches was reduced to 40 mg based on our observation of nocturnal bradycardia on hospitalization day 14 (150 mg P-LAI, 40 mg blonanserin patches: CP-eq. 1,000 mg). Although the patient's bradycardia then disappeared, her psychotic symptoms including her catatonic syndrome became grossly exacerbated, characterized by negativism (such as refusal to eat anything), mutism, and stupor (BPRS, 66 points: positive symptoms, 21 points; negative symptoms, 20 points) on hospitalization day 28.

We administered 13 ECT sessions at 2×/week from hospitalization day 38 to day 87. During the ECT sessions, we added risperidone of 2 mg due to the patient's profound catatonic state, and her psychotic symptoms improved.

After the completion of this ECT regimen, we changed the 150 mg P-LAI and 40 mg blonanserin patches to risperidone in order to reduce the dosage of antipsychotic drugs, since the patient did not accept our proposal of medication with P-LAI alone or with clozapine. On hospitalization day 90, her BPRS score was 27 points (positive symptoms, 5 points; negative symptoms, 3 points) when the risperidone dose was only 7.5 mg (CP-eq. 750 mg). She was discharged to her home on day 128 with the continued 7.5 mg risperidone. Two years after discharge, she remained in remission under treatment with 8 mg risperidone.

## Discussion

Our patient showed a better response to haloperidol during her initial 10 years after the onset of schizophrenia. However, she was subsequently administered a gradual dosing-up of antipsychotics over the course of two relapse episodes, and a very high dose of polypharmacy including LAI was ultimately required to control her continuous psychotic state. During this clinical course, the switch of antipsychotics including aripiprazole resulted in a quick worsening of the patient's psychosis (rebound psychosis), leading to a further increase in the antipsychotic dose. As a result, the severe exacerbation of psychosis necessitated the patient's admission to another hospital, and she met the criteria of TRS; that is, she had developed tolerance to the effects of antipsychotics. Overall, this clinical picture confirmed that the DS state was established in the patient, and she exhibited signs of DSP.

The basic strategy for a patient with an established DS state is to stably block the patient's D2Rs but not provide a blockade that is too excessive, which could mask the appearance of DSP with antipsychotics. This strategy leads to the recommendation of medications with long half-lives (including LAIs) relative to those with short half-lives [2]. However, patients in the DS state are generally administered a high dose of antipsychotic medication (often over CP-eq. 1,000 mg), which cannot be covered with monotherapy even at the authorized maximum dose (i.e., corresponding to CP-eq. 800 mg for paliperidone 12 mg, luransidone 80 mg, and so on). It is thus difficult to switch such a high-dose medication to monotherapy comprising an antipsychotic agent with a long half-life. Notably, pharmacotherapy with a long-half-life antipsychotic such as blonanserin or risperidone long-acting injectable (RLAI) was reported to be effective for DSP in patients with schizophrenia, but these regimens were add-on medications combined with ongoing antipsychotic medication [13,14]. These findings indicate that it can be extremely difficult in such cases to decrease the antipsychotic dose and break away from the established DS state. The use of such an add-on antipsychotic poses a risk of the renewal of DSP episodes as well.

ECT's underlying mechanism of action is not completely understood, but multiple pathways such as the enhancement of GABAergic function, a modulation of serotonergic systems, enhanced neurogenesis, and the release of neurotropic factors have been proposed to be involved in the beneficial effects of ECT [15]. One of the possible mechanisms underlying the effects of ECT is D2R downregulation. This change in the regulation of D2Rs at the receptor level was reported in patients with major depressive disorder [8,9]. In addition, an investigation using an animal model of the DSP model demonstrated that electroconvulsive shocks can reduce the density of D2Rs [10].

Our patient's successful response to the treatment provided at our hospital may indicate that the administration of ECT may resolve the DS state. Although our patient's actual D2R density could not be measured, her subsequent successful maintenance of a stable psychotic state with a lower dose of risperidone than that used earlier indirectly supports the possible downregulation of D2Rs. Since ECT is effective for catatonic state [16], we speculate that our patient's marked improvement might be attributable in part to the effect of the ECT on her catatonia.

This patient's case supports two reports (including one from our research group) showing that the conditions of patients with severe DSP were largely improved with the application of ECT [11,12]. However, the treatment strategies following the use of ECT for those patients differed from the strategy we used for the present patient; in the previous two reports [11,12], once-monthly aripiprazole (AOM) was administered after ECT since AOM, as a dopamine partial agonist, has less potential to cause the DS state [17]. We chose to treat our patient with oral risperidone again after she showed dramatic improvement with the ECT, since we continued the P-LAI during the patient's ECT sessions. This choice was also related to our concern that the DS state could persist at the receptor level despite the disappearance of signs of DSP at the clinical level, where the introduction of AOM still poses a risk of worsened psychosis. As mentioned above, treatment with relatively high-dose risperidone can result in additional DSP in the future. Thus, a further switch to a dopamine partial agonist or a repeated introduction of LAI may be effective in similar cases.

## Conclusions

Clozapine is the gold standard for patients who meet the criteria for TRS, as our patient did. However, clozapine is not widely used because of its serious potential adverse effects (e.g., agranulocytosis, myocarditis, and seizures) and the necessary cumbersome blood monitoring, both of which lead to some hesitation among clinicians and patients. The management of patients with TRS for whom the clozapine option is unavailable is difficult in clinical practice, but the observation of signs of DS or DSP provides clinicians with useful clues regarding the selection of an optimal treatment plan.

Although our search of the relevant literature identified few case reports focusing on the efficacy of ECT against DSP and TRS, our patient's case demonstrates that ECT can resolve TRS symptomatology related to DS or DSP. However, strategies for the treatment of DSP and a resolution of the DS state are still at an undeveloped stage. Since resolving the DS state provides efficacy and safety benefits in terms of the pharmacological treatment of DSP, it is necessary to accumulate a large number of cases of patients with schizophrenia who have DSP and whose symptoms are improved by ECT combined with a reduction of the maintained antipsychotic dose. An accumulation of data regarding the use of ECT for patients who have TRS but cannot be treated with clozapine is desired.
